# Virtual screening for plant PARP inhibitors – what can be learned from human PARP inhibitors?

**DOI:** 10.1186/1758-2946-4-S1-O24

**Published:** 2012-05-01

**Authors:** Peter-Paul Heym, Wolfgang Brandt, Ludger A Wessjohann, Hans-Joachim Niclas

**Affiliations:** 1 Leibniz-Institute for Plant Biochemistry, Halle, 06114, Germany; 2 Agrochemical Institute Piesteritz e.V. (AIP), Wittenberg, 06886, Germany

## 

The functions of Poly(ADP-ribose) polymerase enzymes (PARPs) in general are best studied based on human PARP-1 (*Hs*PARP-1). *Hs*PARP-1 is well investigated because pharmacological modulation of its activity modulates DNA-binding of antitumor drugs [1]. In contrast to human PARP enzymes, the exact role of PARPs in plants remains to be elucidated. Different stresses activate plant PARP enzymes to mediate DNA repair and (programmed) cell death whereas the addition of PARP inhibitors decreases the degree of cell death [2]. Therefore, the development of plant PARP inhibitors might be a way to increase the tolerance against abiotic stress.

Initial to searches in commercial databases for potential plant PARP inhibitors, a virtual screening route had to be established for human PARP-1 inhibitors. Simultaneously, every step in that procedure was applied on a plant PARP enzyme to investigate the differences of both active sites. All differences have been evaluated statistically, e.g. using receiver-operator characteristics (ROC) and power analyses. At the end of that parallel screening route, a docking threshold for *Arabidopsis thaliana* L. PARP-1 (*At*PARP-1) could be derived by knowledge transfer from the corresponding human receptor and its inhibitors.

**Figure 1:**
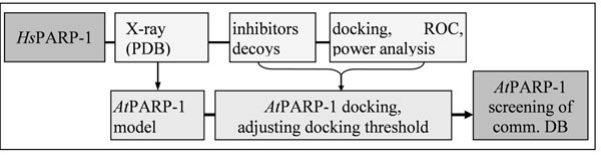
Key steps in virtual screening routes for human and *Arabidopsis thaliana* L. PARP-1. The results have been used to successfully apply the screening process for *At*PARP-1 on a commercial database.

Knowing the differences of the human and plant screening routes, predictions of the applicability of that multi-step process on a commercial database have been explored. Finally, the developed virtual screening route has been applied to screen a commercial database for *At*PARP-1 inhibitors. From 20 compounds tested so far *in vitro*, 13 show inhibitory effects.
